# Yeast-Derived Glucan Particles: Biocompatibility, Efficacy, and Immunomodulatory Potential as Adjuvants and Delivery Systems

**DOI:** 10.3390/pharmaceutics17081032

**Published:** 2025-08-08

**Authors:** João Panão-Costa, Mariana Colaço, Sandra Jesus, Filipa Lebre, Maria T. Cruz, Ernesto Alfaro-Moreno, Olga Borges

**Affiliations:** 1 CNC-UC—Center for Neuroscience and Cell Biology, University of Coimbra, 3004-504 Coimbra, Portugal; jpanao94@gmail.com (J.P.-C.); mariana.colaco.139722@gmail.com (M.C.); sjesus.mg@gmail.com (S.J.); trosete@ff.uc.pt (M.T.C.); 2 CIBB—Center for Innovative Biomedicine and Biotechnology, University of Coimbra, 3004-504 Coimbra, Portugal; 3 Faculty of Pharmacy, University of Coimbra, 3000-548 Coimbra, Portugal; 4 Nanosafety Group, International Iberian Nanotechnology Laboratory, 4715-330 Braga, Portugal; filipa.lebre@inl.int (F.L.); ernesto.alfaro@inl.int (E.A.-M.)

**Keywords:** β-glucan, immunotoxicity, hepatitis B vaccine, HBsAg, HBcAg, CL097, *Saccharomyces cerevisiae*, dendritic cell, peripheral blood mononuclear cells, cellular immunotherapy

## Abstract

**Background/Objectives**: Glucan particles (GPs), derived from *Saccharomyces cerevisiae* yeast, possess unique biomedical properties. Nevertheless, it is imperative that a comprehensive risk assessment is conducted during pre-clinical development. GPs are primarily constituted of a naturally occurring polymer known as β-glucan. This study characterized GPs, focusing on physicochemical attributes, biocompatibility, and immunomodulatory potential. **Methods**: GPs were characterized for size, morphology, surface charge, and protein encapsulation efficiency using dynamic light scattering (DLS), electron microscopy, and encapsulation assays. Biocompatibility was assessed through cytotoxicity assays (MTT), hemolysis tests, and measurement of reactive oxygen (ROS) and nitric oxide (NO) production in immune cells. Immunomodulatory potential was evaluated by cytokine and chemokine secretion analysis in peripheral blood mononuclear cells (PBMCs) and monocyte-derived dendritic cells (moDCs) and through in vivo immunization studies in a murine model, focusing on cellular immune responses. **Results**: GPs demonstrated stable physicochemical properties and efficient protein encapsulation, highlighting their suitability as vaccine delivery systems. They exhibited biocompatibility by not inducing cytotoxicity, hemolysis, or excessive ROS and NO production. In PBMCs, GPs stimulated cytokine secretion, suggesting their adjuvant potential. GPs were efficiently internalized by monocytes and led to specific chemokine secretion in stimulated moDCs. In a murine model, GPs induced distinctive cellular immune responses, including TNF-α and IFN-γ production and effector memory T cell activation. **Conclusions**: These findings emphasize GPs’ biocompatibility and immunomodulatory effects, highlighting their potential in immunotherapy and vaccine development, particularly for targeting infectious agents like hepatitis B virus.

## 1. Introduction

As we strive for global disease eradication, the development of improved vaccination strategies is vital. Enthusiasm for particulate systems has risen in recent years, specifically in the field of vaccine development. Using particulate systems as vaccine delivery agents offers numerous advantages, including enhanced bioavailability, controlled release kinetics, and targeted delivery capabilities [1]. Additionally, certain materials possess inherent immune-modulating properties, which can effectively influence and modulate both cellular and humoral immune responses [2]. Leveraging the dual functionality of particulate systems as both delivery vehicles and adjuvants is believed to significantly enhance the immunological outcomes of vaccination.

Despite the growing number of studies on particulate-based vaccine delivery systems, their adverse effects on the organism are not often mentioned or accounted for [3]. Given the unique properties of particulate delivery systems, it is critical to perform a thorough risk assessment during pre-clinical development to unravel their unexpected toxicity profile and to prevent any hazardous reaction on humans [4]. The resulting side effects may derive from the delivery systems’ physicochemical characteristics, leading to unpredictable outcomes from their interaction with the immune system [5,6]. Thus, in vitro assays are crucial to identify valuable candidates and to anticipate any toxicological issues, expediting their potential translation into the clinic [3,4].

β-glucans are natural polymers found in many organisms such as fungi, bacteria, and some algae. These biomaterials are “generally regarded as safe” (GRAS) substances and they are already used in some oral nutraceuticals [7]. β-glucans can enhance immunity and act as immunomodulators, thus being considered biological response modifiers [8]. Based on their immunomodulatory potential, some of the most commonly studied β-glucans are β-1,3-glucans from fungal cell walls, which can have varying β-1,6 branching patterns and sizes [9]. These molecules can behave as pathogen-associated molecular patterns (PAMPs) that bind to pattern recognition receptors (PRRs) at the surface of antigen-presenting cells (APCs), such as dendritic cells (DCs) and macrophages [10]. When glucans are recognized by PRRs like TLR 2/6 and Dectin-1, an intracellular signalling cascade is triggered, promoting the expression of various molecules responsible for the regulation of both innate and adaptive immunity [11].

Glucan particles (GPs) are porous and hollow cell wall microspheres with a diameter of 2–5 µm [12]. They are generally purified from the baker’s yeast, *Saccharomyces cerevisiae*, through successive alkaline, acidic and organic extractions at high temperatures, resulting in a final GP mainly composed of β-1,3-D-glucan and trace amounts of mannoproteins and chitin [12,13,14]. Since their origin, GPs are often tested as particulate delivery systems for several types of molecules, such as proteins, DNA, siRNA, and even nanoparticles [12,15,16,17]. Due to their β-glucan content and other pathogen-mimicking characteristics, GPs are targeted to phagocytic cells, like DCs and macrophages, making them interesting candidates to use in immunotherapies or as a vaccine platform [18]. Alongside their use as a payload delivery system, these yeast-derived β-glucan particles have already been studied for their potential adjuvant and immunostimulant effects, namely in hepatitis B virus (HBV) vaccination. In light of the challenge posed by inadequate immune responses in clearing HBV infections, the use of GPs as both immunomodulatory agents and delivery systems represents a compelling strategy [19].

In the context of HBV infection, formulating a dual-antigen vaccine that incorporates both the surface antigen (HBsAg) and the core antigen (HBcAg) presents several advantages, mainly by stimulating both humoral and cellular immune responses [20]. Additionally, Toll-like receptors (TLRs) 7 and 8, known agonists that recognize viral single-stranded RNA, are able to initiate signalling pathways contributing to immune cell activation [21]. Of particular interest, CL097, a water-soluble small molecule compound with selective TLR7 activation capabilities, holds the potential to enhance vaccine efficacy [22].

This current investigation sets out to comprehensively assess the physicochemical attributes of GPs while simultaneously evaluating their toxicity, biocompatibility, and immunomodulatory effects. Furthermore, our aim is to determine the efficacy of GPs in potentiating the immune response against HBV. This is achieved by examining the immune response triggered through vaccination with GPs encapsulating HBsAg, HBcAg, and the TLR7 agonist CL097, collectively referred to as GPs (HHC). By exploring the potential of GPs as vaccine delivery candidates and exploring their immunotoxicological profiles, this study not only contributes to the development of innovative strategies for enhancing immune responses against HBV but also sheds light on the importance of biocompatibility assessment of particles soon to be integrated in the medical field.

## 2. Materials and Methods

### 2.1. Materials

Fermipan Red Instant Dried Yeast—*Saccharomyces cerevisiae*—was sourced from Equipan (Antanhol, Portugal). The RAW 264.7 murine cell line, Lipopolysaccharides from *Escherichia coli* 055:B5 (LPS), MTT (3-[4,5-dimethylthiazol-2-yl]-2,5-diphenyl tetrazolium bromide), torula yeast type IV (tRNA), Concavalin A, and sodium pyruvate solution were all obtained from Sigma-Aldrich Corporation (St. Louis, MO, USA). Apyrogenic water was generously provided by Fresenius-Kabi (Campo de Besteiros, Portugal). RPMI 1640 Medium (Cat. Number: 42401018), MEM Amino Acids Solution, MEM Non-Essential Amino Acids Solution, GlutaMAX, and DCFH-DA (2′,7′-dichlorodihydrofluorescein diacetate) were purchased from Thermo Fisher Scientific Inc. (Waltham, MA, USA). Recombinant Human IL-4 and GM-CSF, as well as Human TNF-α, Human IL-6, and Murine TNF-α ELISA kits, were acquired from PeproTech (Rocky Hill, NJ, USA). Monoclonal antibodies including anti-mouse CD4 FITC, anti-mouse IFN-γ PE, anti-mouse TNF-α PerCP/Cyanine5.5, anti-mouse CD8a APC, anti-mouse/human CD44 PE and anti-mouse CD62L PerCP were obtained from Biolegend (San Diego, CA, USA). Recombinant Hepatitis B Surface Antigen (adw) (DAG3942) and Recombinant HBV Core Antigen (DAG593) were sourced from Creative Diagnostics (Shirley, NY, USA). IgG HRP was acquired from Bethyl Laboratories Inc. (Montgomery, TX, USA). All other reagents used in this study were of analytical grade and were procured from standard suppliers.

### 2.2. Methods

#### 2.2.1. GPs’ Purification

Glucan particles (GPs) were derived from dried *Saccharomyces cerevisiae* yeast using a modified method as outlined by Soto et al. [12], with the aim of obtaining hollow and porous cell walls predominantly composed of β-glucans. Briefly, 20 g of dried yeast was suspended in 200 mL of 1 M NaOH and heated to 85 °C for 1 h with continuous rotational stirring. Following centrifugation at 3000× *g* for 10 min, the insoluble yeast cell walls were collected. This process was repeated twice, first for an additional hour and then for 10 min. The resultant pellet was resuspended in 200 mL of ultra-pure water, with pH adjustment to 4.5 using an HCl solution. The suspension was subjected to rotational stirring for 1 h at 75 °C. Subsequently, the insoluble material was retrieved via centrifugation and successively washed three times with 200 mL of ultra-pure water, followed by three washes with 40 mL of isopropanol and, ultimately, two washes with 40 mL of acetone. The ultimate insoluble material obtained was allowed to air-dry at room temperature.

#### 2.2.2. Protein and CL097 Encapsulation

Protein loading into GPs followed the protocol described by De Smet et al. [23] with minor adaptations. Initially, 10 mg of GPs were swollen with 100 µL of protein/CL097 solution in 0.9% saline at 4 °C for 2 h. The swollen GPs were subsequently frozen at −80 °C and subjected to overnight freeze-drying. The resultant powder was combined with 100 µL of 25 mg/mL torula yeast RNA dissolved in TEN buffer (50 mM Tris-HCl, pH 8, 2 mM EDTA, 0.15 M NaCl) and incubated for 30 min at 50 °C. Following this step, 450 µL of 10 mg/mL tRNA was introduced, and the mixture was incubated for an additional hour at 50 °C. The GPs were then subjected to three rounds of washing through centrifugation (10 min, 2000× *g*) with the inclusion of 500 µL of 0.9% saline at each washing step. Finally, the pellet of GP was resuspended in 1 mL of 0.9% saline. Supernatants derived from ovalbumin, myoglobin, and lysozyme were collected and maintained at −20 °C until analysis via SDS-PAGE. Importantly, this method can be readily scaled down while preserving appropriate ratios. The loading efficiency (LE) and loading capacity (LC) of GPs were estimated based on the amount of unencapsulated protein remaining in the supernatants and washes after the encapsulation process. An indirect qualitative method using SDS-PAGE was applied. Known concentrations of ovalbumin were run in parallel as standards to approximate the detection threshold and visually estimate the residual unencapsulated protein in experimental samples.

(1) $$Loading\;Efficiency\left(LE\;\%\right)=\frac{\left(Total\;protein\;added-Unencapsulated\;protein\right)}{\left(Total\;protein\right)}\times 100$$

(2) $$Loading\;Capacity\left(LC\;\%\right)=\frac{\left(Encapsulated\;protein\right)}{\left(Total\;protein\right)}\times 100$$

#### 2.2.3. Size and Zeta Potential

The size, polydispersity index (PDI), and zeta potential of GPs were assessed utilizing a Zetasizer Nano-ZS device (Malvern Instruments Ltd., Malvern, UK). The particle size was ascertained via dynamic light scattering (DLS), while the zeta potential was determined by employing electrophoretic light scattering (ELS). These measurements adhered to established procedures outlined for the Zetasizer Nano-ZS system. GPs were measured in water, 0.9% NaCl, DMEM and RPMI cell culture medium. To assess the colloidal stability of GPs, particle size and PDI were measured over time in the same media. The dispersions were incubated at 37 °C for 24 h, 48 h, and 168 h. At each time point, samples were brought to room temperature prior to measurement, and particle size and PDI were analyzed.

#### 2.2.4. Protein Electrophoresis

Protein encapsulation within GPs was assessed through sodium dodecyl sulphate polyacrylamide gel electrophoresis (SDS-PAGE) analysis, utilizing the first supernatant collected during the protein loading process. Samples were blended in a 1:4 *v*/*v* ratio with denaturing loading buffer (comprising 4% SDS, 20% glycerol, 10% 2-mercaptoethanol in 0.125 M Tris buffer at pH 6.8, and 0.004% bromophenol blue as a colour marker) and then incubated at 95 °C for 5 min. The resultant samples were subsequently subjected to separation on polyacrylamide gels fabricated from a 40% Acrylamide solution and a 2% Bis-acrylamide solution (Bio-Rad, Hercules, CA, USA). For visualization of distinct protein content post-electrophoresis, the polyacrylamide gels were immersed in a 0.25% Coomassie blue R-250 solution (Fluka chemical corp., Milwaukee, WI, USA) for 30 min. Subsequently, excess staining was eliminated by treating the gels with a destaining solution consisting of 7.5% methanol and 10% acetic acid.

#### 2.2.5. Transmission Electron Microscopy (TEM)

TEM images were acquired utilizing a JEOL JEM 1400 microscope operating at 120 kV (JEOL, Peabody, MA, USA). During sample preparation, GPs were suspended in ultra-pure water. Subsequently, a droplet of this suspension was deposited onto a mesh grid and allowed to dry prior to observation.

#### 2.2.6. Raw 264.7 Cell Line

The RAW 264.7 murine macrophage cell line was cultivated following standard protocols at 37 °C with 5% CO_2_. The growth medium consisted of Dulbecco’s Modified Eagle’s Medium (DMEM) supplemented with 10% heat-inactivated fetal bovine serum (FBS), 1% Penicillin/Streptomycin, 10 mM HEPES, and 3.7 g/L sodium bicarbonate. Subsequent subculturing was performed upon reaching approximately 80% confluence. This involved mechanically detaching the cells with a cell scraper and diluting them at ratios ranging from 1:2 to 1:6, conducted every 2 or 3 days. Notably, cells used in these experiments were utilized up to the 20th passage.

##### Uptake Studies by Flow Cytometry and TEM

RAW 264.7 macrophages were seeded into a 48-well plate at a density of 1 × 10^5^ cells/well and allowed to adhere overnight. Subsequently, the culture medium was substituted with serum-free DMEM, and the cells were exposed to GPs (BSA-FITC) at a concentration of 5 µg/mL for a duration of 4 h. After incubation, the cells were gently detached by mechanical means. The harvested cells were then rinsed using a 1% FBS PBS solution, followed by fixation with 2% paraformaldehyde for a period of 20 min. Flow cytometric analysis was conducted using a BD Accuri C6 instrument.

For TEM visualization, cells were collected by centrifugation and fixed with 2.5% glutaraldehyde in 0.1 M sodium cacodylate buffer (pH 7.2) for 2 h. After rinsing with the same buffer, post-fixation was conducted using 1% osmium tetroxide for 1 h. Subsequently, the samples were washed with distilled water and dehydrated using an ethanol gradient series (70% to 100%). The cell pellets were then embedded in 2% molten agar, further dehydrated in ethanol (30–100%), impregnated, and finally embedded in epoxy resin. Ultra-thin sections were prepared, mounted on copper grids, and stained with 0.2% lead citrate for 7 min. Observations were performed using an FEI Tecnai G2 Spirit BioTWIN microscope operating at 100 kV.

##### Cell Viability Assay

RAW 264.7 cells were seeded into 96-well plates at a density of 2 × 10^4^ cells/well and allowed to incubate overnight. Subsequently, the growth medium was substituted with fresh medium along with the respective test formulations, and the cells were further incubated for a duration of 24 h. Following this incubation period, 20 µL of MTT solution (5 mg/mL) was introduced into each well. Following a 90-minute incubation, the supernatants were aspirated, and 100 µL of dimethyl sulfoxide (DMSO) was added to dissolve the formazan crystals that had developed. Subsequently, the absorbance of the wells was measured at both 540 nm and 630 nm using a microplate reader. The relative cell viability (%) was subsequently calculated by comparing the absorbance values with those of the negative control, according to Equation (3).

(3) $$Cell\;viability\left(\%\right)=\frac{\left(OD\;sample\;\left(540\;nm\right)-OD\;sample\;(630\;nm)\right)}{\left(OD\;control\;\left(540\;nm\right)-OD\;control\;(630\;nm)\right)}\times 100$$

##### Reactive Oxygen Species (ROS) Production Assay

RAW 264.7 cells were seeded into black 96-well plates at a concentration of 5 × 10^4^ cells/well, and these plates were incubated overnight. Post-incubation, the culture medium was substituted with fresh medium (150 μL) and test formulations (50 μL), and the cells were subjected to a 24 h incubation. LPS served as a positive control and was applied at 1 μg/mL. Subsequent to the incubation period, the supernatants were aspirated, and 200 µL of serum-free DMEM containing 50 µM DCFH-DA (dichlorofluorescein diacetate) was introduced to each well. This configuration was allowed to incubate for a duration of 2 h, during which fluorescence intensity was gauged at excitation/emission wavelengths of 485/20 nm and 528/20 nm. To ascertain the fold increase in fluorescence, a comparison was conducted between the fluorescence intensity and that of the negative control according to Equation (4). Subsequently, the cell culture was discarded, and the MTT reduction assay was conducted for the assessment of cell viability (Equation (3)).

(4) $$ROS\;Production=\frac{{Fluorescence}_{SAMPLE}}{{Fluorescence}_{NEGATIVE\;CONTROL}}$$

##### LPS-Spiked ROS Production Assay

This protocol closely resembles the ROS production assay, with the exception of the step involving sample addition. In this variation, subsequent to the initial incubation and removal of the medium, each well received 100 μL of fresh DMEM. To this, 50 μL of an aqueous suspension of GPs and 50 μL of LPS were introduced (final concentration: 1 μg/mL). ROS production and cell viability were calculated according to Equation (4) and Equation (3), respectively.

##### Nitric Oxide (NO) Production Assay

RAW 264.7 cells were seeded into a 48-well plate at a concentration of 2.5 × 10^5^ cells/well and allowed to incubate overnight. Following this, the culture medium was exchanged for fresh medium (375 μL) in conjunction with the test formulations (125 μL), and the cells underwent a 24 h incubation. LPS was utilized as a positive control, applied at 1 μg/mL. After the incubation period, 100 µL of supernatant from each well was transferred into a separate 96-well plate. Subsequently, the residual cell culture was discarded, and the MTT reduction assay was executed to ascertain cell viability (Equation (3)). In order to quantify NO production, a calibration curve was established within the same 96-well plate. This involved utilizing various dilutions of a 1 mg/mL NaNO_2_ stock solution in DMEM without phenol red, spanning concentrations of 0.625, 1.25, 2.5, 5, 10, 20, 30, 50, 60, 70, and 80 µM of NaNO_2_. Following this, 100 µL of Griess reagent was introduced into each sample, as well as into the calibration curve. The plate was then incubated in the dark, at room temperature, for 10 min. Subsequent to this incubation, absorbance was measured at 550 nm. The Griess reagent was prepared by immediate mixing of equal volumes of 1% (*w*/*v*) sulphanilamide in 2.5% (*v*/*v*) phosphoric acid and 0.1% (*w*/*v*) naphthylethylenediamine dihydrochloride in 2.5% (*v*/*v*) phosphoric acid just before use. NO production was calculated according to Equation (5).

(5) $$NO\;Production\left(\%\right)=\frac{{NO\;(µg/mL)}_{SAMPLE}}{{NO\;(µg/mL)}_{POSITIVE\;CONTROL}}\;\times 100$$

##### LPS-Spiked NO Production Assay

This procedure closely resembles the NO production assay, with the exception of the step involving sample addition. In this modified version, subsequent to the initial incubation and removal of the medium, each well received 250 μL of fresh DMEM. Additionally, 125 μL of an aqueous suspension of GPs and 125 μL of LPS (final concentration: 1 μg/mL) were introduced. NO production and cell viability were calculated according to Equation (5) and Equation (3), respectively.

#### 2.2.7. Hemolysis Assay

Fresh whole blood was obtained from healthy human donors and collected into lithium heparin tubes, in compliance with the pre-established protocol of the Laboratory of Clinical Analysis at the Faculty of Pharmacy, University of Coimbra. Ethical considerations were followed, and written informed consent was acquired from all participants, following the principles outlined in the Declaration of Helsinki. The collected blood was appropriately diluted to establish a total hemoglobin concentration of 10 mg/mL, ensuring that the absorbance values of the samples fell within the calibration range. In a microtube, 100 μL of formulation at varying concentrations was mixed with 700 μL of PBS and 100 μL of diluted blood. To evaluate potential interferences from GPs, all samples were subjected to the same concentration of incubation with PBS alone. As a positive control (PC), Triton-X-100 at 1% was utilized, while PBS was employed as a negative control (NC). These microtubes were incubated at 37 °C for 3 h, with periodic shaking every 30 min. After incubation, the tubes were subjected to centrifugation at 800× *g* for 15 min. In a 96-well plate, 100 μL of CMH reagent was added to 100 μL of the supernatant from PC, NC, and sample tubes. The plate was shaken for 3 min, and absorbance was measured at 540 nm using a microplate reader. The percentage of hemolysis was calculated using the following equation (Equation (6)):

(6) $$Hemolysis\left(\%\right)=\frac{({OD}_{SAMPLE}\;-\;{OD}_{NEGATIVE\;CONTROL})\;}{\left({OD}_{POSITIVE\;CONTROL}\;-\;{OD}_{NEGATIVE\;CONTROL}\right)}\times 100$$

#### 2.2.8. Human PBMCs’ Isolation, Monocyte Purification, and moDCs’ Differentiation

Peripheral blood (buffy coat) was generously contributed by IPST, IP (Coimbra, Portugal), and was sourced from healthy donors who had provided written informed consent. All procedures adhered to institutional ethical guidelines and received approval from the relevant ethics committee (approval number CE-063/2019). PBMCs were isolated through density gradient separation using Lymphoprep (Axis-Shield, Dundee, Scotland), following the manufacturer’s protocol with slight modifications. Initially, the blood was diluted with 0.9% sodium chloride at a ratio of 2:1 (*v*/*v*). Subsequent centrifugation at 1190× *g* for 20 min at 20 °C (without brake) enabled the collection of the dense mononuclear cell ring. This ring underwent two washes with PBS (pH 7.4 at 37 °C) via centrifugation (350× *g*, 10 min, 20 °C). Finally, the cells were suspended in RPMI 1640 medium supplemented with 1% Penicillin/Streptomycin, 20 mM HEPES, 2 mM L-glutamine, and 10% heat-inactivated FBS. PBMCs’ viability was determined using trypan blue exclusion, and only cells with a viability surpassing 90% were utilized for subsequent experiments.

Monocytes were isolated from human PBMCs using the MidiMACS™ Separator with magnetic human CD14 MicroBeads (Miltenyi Biotec, Bergisch Gladbach, North Rhine-Westphalia, Germany). The isolated monocytes were cultured in 6-well plates at a concentration of 1.3 × 10^6^ cells/mL in 3 mL of DC medium. The DC medium consisted of RPMI 1640 (GibcoTM) supplemented with 1% Penicillin/Streptomycin, 25 mM HEPES, 10% heat-inactivated FBS, 2 mM L-glutamine, 0.1 mM MEM Non-Essential Amino Acids Solution, and 1 mM sodium pyruvate.

To induce moDC differentiation, IL-4 (50 ng/mL) and GM-CSF (40 ng/mL) were introduced. The plates were incubated over the course of 6 days, with a medium replacement on day 3. On day 6, the moDCs were detached from the wells, collected, and centrifuged at 350× *g* for 5 min at room temperature. The resulting concentrated immature moDCs were suspended in DC cell culture media without supplementation. Subsequent cell counting and assessment of cell viability were performed using trypan blue exclusion.

#### 2.2.9. Proliferation Assay in Human PBMCs

Human PBMCs were plated into 96-well plates at a density of 5 × 10^5^ cells/well and were allowed to incubate for 2 h. Following this, the culture medium was substituted with fresh medium along with the respective test formulations, and the cells were incubated for a duration of 72 h. After the completion of the incubation period, 20 µL of MTT solution (5 mg/mL) was introduced into each well. Following a 4 h incubation, the plate underwent centrifugation at 800× *g* for 25 min, following which the supernatant was meticulously removed. Subsequently, formazan crystals were dissolved using 100 µL of DMSO, and the absorbance was gauged at 540 nm and 630 nm using a microplate reader. Cell viability (%) was then calculated according to Equation (3).

#### 2.2.10. Cytokine Quantification in Human PBMCs

Human PBMCs were seeded into 96-well plates at a concentration of 2.5 × 10^4^ cells/well and incubated for 24 h. Following this, the culture medium was exchanged for fresh medium along with the designated test formulations, and the cells were incubated for an additional 24 h. Subsequently, supernatants were collected and stored at −80 °C for subsequent analysis. For the assessment of TNF-α and IL-6 release, the ELISA technique was employed in accordance with the instructions provided by the kit manufacturer (Peprotech).

#### 2.2.11. Uptake Studies in Human Monocytes Through Confocal Laser Scanning Microscopy (CLSM)

BSA-FITC (100 μL of a 1 mg/mL aqueous solution) was encapsulated into 10 mg of GPs following the procedure detailed in Section 2.2.2. Human monocytes were seeded onto glass coverslips within 24-well plates (5 × 10^6^ cells/mL, 250 μL) and cultivated for 2 h, facilitating monocyte attachment. Subsequent to attachment, the cell culture medium was replaced, and GPs (BSA-FITC) were introduced into each well at a concentration of 50 μg/mL. Cells were then subjected to incubation periods of 2 h, 6 h, 24 h, 48 h, or 72 h. Following each uptake period, the medium was removed, cells were washed using PBS (pH 7.4) and subsequently fixed with 4% paraformaldehyde in PBS. Following fixation, human monocytes underwent three washes with PBS, after which they were stained with Hoechst 33342 (Nucleus) and Alexa Fluor™ 594 (Plasma membrane). Following staining, cells were washed twice with PBS, mounted with coverslips onto microscope slides using DAKO mounting medium, and then visualized under a Carl Zeiss LSM 710 confocal point-scanning microscope.

#### 2.2.12. Cytokine and Chemokine Quantification in Human moDCs

MoDCs were seeded into a 24-well plate at a density of 2 × 10^5^ cells/well and were allowed to incubate overnight. Following this, GPs (2.5 µg/mL) were introduced to the wells, adjusting the final volume to 1 mL per well. Incubation was sustained for 24 h, after which supernatants were collected by centrifugation at 500× *g* for 3 min and stored at −80 °C. To ascertain the levels of cytokines and chemokines released by moDCs, the collected supernatants underwent assessment utilizing the Cytokine and Chemokine 34-Plex Human ProcartaPlex™ Panel 1A (Catalog number: EPX340-12167-901), adhering to the guidelines outlined by the manufacturer.

#### 2.2.13. In Vivo Immunization Study

Five-week-old female C57BL/6 mice were obtained from Charles River Laboratories (Barcelona, Spain). Following their arrival, the mice were allowed a one-week acclimation period in the local animal house facility. They were housed under standard conditions adhering to a 12 h light/dark cycle and were provided with unrestricted access to food and water. All procedures involving animals were conducted in accordance with institutional ethical guidelines and received approval from the appropriate ethics committee (approval number DGAV 0421/000/000/2020). These studies were also conducted in alignment with relevant national regulations (Dec. No. 113/2013) and international legislation (2010/63/EU Directive) governing the utilization of animals in research. Vaccination of the mice was carried out utilizing saline water as the vehicle in both the HBsAg and HBcAg (HH) vaccination group and GPs encapsulated with HBsAg, HBcAg and CL097 (HHC) vaccination group. The vaccination regimen encompassed two subcutaneous (SC) administrations (100 µL/dose). The study culminated on day 21, when the mice were euthanized via cervical dislocation. Antigens and CL097 were encapsulated into GPs following the procedure detailed in Section 2.2.2. Information regarding the vaccination groups and schedule is outlined in Table 1.

##### Blood Collection

Blood samples were obtained on day 21 via submandibular venipuncture using an animal lancet. The collected blood was allowed to coagulate over a span of 4 h. After coagulation, the samples underwent centrifugation at 4500× *g* for 10 min, facilitating the separation of serum. The supernatant obtained post-centrifugation was meticulously transferred to another microcentrifuge tube and stored at −20 °C until it was ready for subsequent analysis.

##### Quantification of Serum Total IgG

For the determination of serum IgG titers specific to HBsAg and HBcAg, an ELISA was employed, following a well-established protocol developed by our research group [24]. In brief, high-binding 96-well plates (Nunc MaxiSorp™ flat-bottom, Thermo Fisher Scientific Inc., Waltham, MA, USA) were coated with 100 µL of 1 µg/mL HBsAg or HBcAg in a solution containing 50 mM sodium carbonate/bicarbonate (pH 9.6). Following a 2 h incubation at 37 °C, the plates were subjected to five washes with PBS-T (PBS with 0.05% Tween 20^®^) and then blocked with 1% BSA in PBS-T for 1 h at 37 °C. After the blocking step, the plates were washed once more, and 100 µL of serum samples (serially diluted starting at 1:64) was added to the wells. Subsequently, the plates were incubated at 37 °C for 2 h. After further washing, 100 µL of HRP-conjugated goat anti-mouse IgG (dilution: 1:10,000) in PBS-T was introduced to each well and incubated for 30 min at 37 °C. The next step involved detection, wherein 100 µL of citrate buffer containing o-phenylenediamine (OPD) and H_2_O_2_ was added to each well. The plates were then incubated for 10 min at room temperature in darkness. The enzymatic reaction was halted by the addition of 50 µL of 1 M H_2_SO_4_, and absorbance was measured at 492 nm. The serum IgG titers were ascertained as the end-point titer. This end-point titer corresponds to the antilog of the last log2 dilution where the optical density (OD) exhibited at least a two-fold increase compared to the value of the equally diluted naive sample. The log2 endpoint titers were employed to normalize the data, thereby minimizing variability.

##### Spleen Cell Isolation

On day 21, spleens were collected, and a single-cell suspension was prepared utilizing a 70 µm cell strainer. The cells were subjected to a single wash by centrifugation at 220× *g* for 10 min, following which they were suspended in 0.5 mL of cold ACK buffer for 1 min. This step served to eliminate red blood cells from the cell culture medium. Subsequently, the cells were diluted and washed with PBS. Spleen cells were then resuspended in a cell culture medium composed of RPMI 1640, 10% heat-inactivated fetal bovine serum (HI-FBS), 1% Penicillin/Streptomycin (PenStrep), 0.05% of 100 mM 2-mercaptoethanol, 1% GlutaMAX, 1% sodium pyruvate, 1% MEM non-essential amino acids, and 2% MEM amino acids.

##### Cytokine Quantification

Subsequent to spleen isolation, 50 µL of the cell suspension was dispensed into a 96-well plate at a density of 1 × 10^7^ cells/mL. To these wells, 125 µL of medium and 25 µL of medium, HBsAg (40 µg/mL), HBcAg (40 µg/mL), or Con A (50 µg/mL) (Con A was used as positive control). The plates were subsequently incubated for a duration of 96 h. This incubation aimed to stimulate cytokine production by antigen-specific cells. Following the incubation period, the supernatants were harvested and stored at −80 °C for future analysis. The concentration of TNF-α was determined using the murine development ELISA kit, in accordance with the instructions provided by the manufacturer (Peprotech).

##### Flow Cytometry Analysis

After spleen isolation, 100 µL of the cell suspension was placed in U-bottom 96-well plates at a concentration of 1 × 10^7^ cells/mL. The plates were then incubated overnight. Following the incubation, 50 µL of purified anti-mouse CD28 Antibody (Biolegend) at 8 µg/mL and 50 µL of either HBsAg or HBcAg at 40 µg/mL were introduced to the cells. This incubation lasted for 1 h. After this period, 10 µL of a 21X Brefeldin A solution was added to each well, followed by an additional 5 h incubation. Subsequent to the incubation, the cells were collected for flow cytometry analysis. They were centrifuged at 350× *g* for 5 min and resuspended in PBS containing 1% FBS. The cells were then stained for 30 min at 4 °C using combinations of CD4/IFN-γ/TNF-α/CD8 or CD4/CD44/CD62L/CD8 in accordance with the manufacturer’s instructions. Following staining, the cells were analyzed using a BD Accuri™ C6 cytometer (BD Biosciences, San Jose, California, USA).

#### 2.2.14. Statistical Analysis

The results were presented as the mean ± standard error of the mean (SEM). Data analysis was conducted using GraphPad Prism software, with GraphPad Prism 8 (GraphPad Software, Inc., La Jolla, CA, USA).

## 3. Results and Discussion

### 3.1. Physicochemical Characterization of GPs: Insights into Size, Surface Charge and Protein Encapsulation

**Figure 1:**
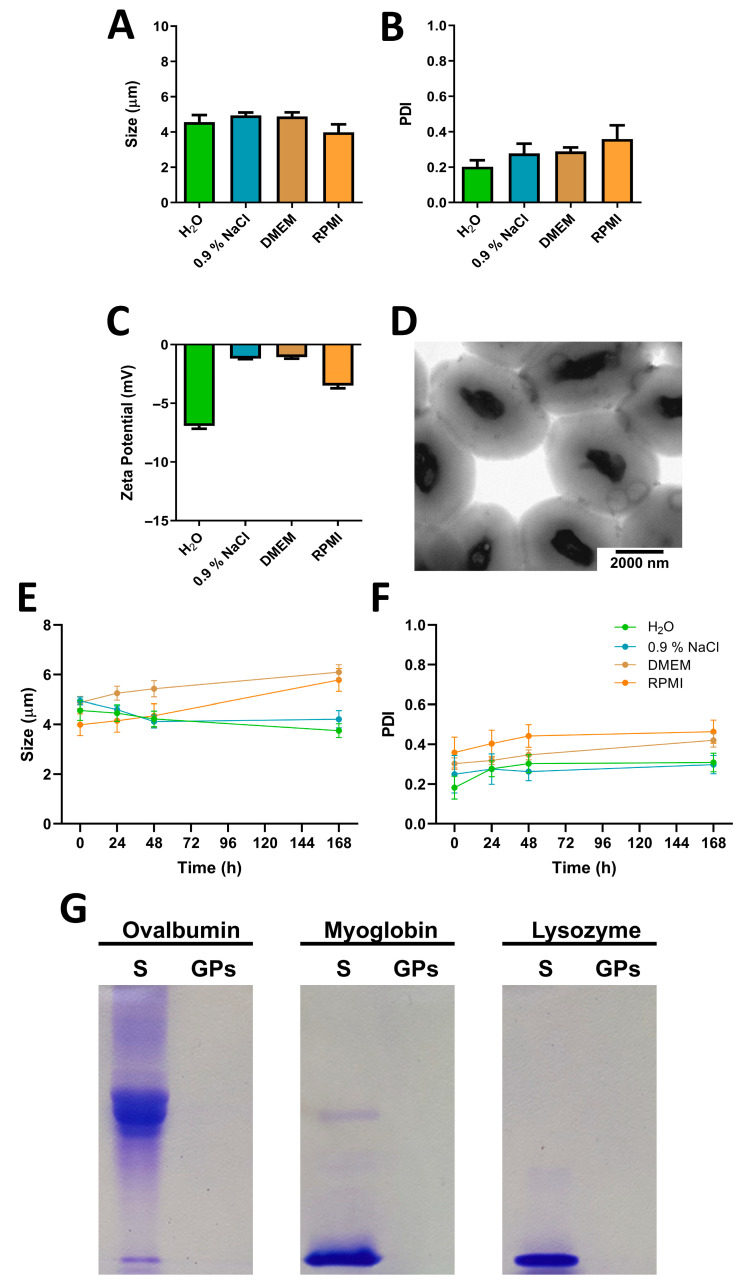
Characterization of glucan particles (GPs). (**A**) Mean particle size, (**B**) PDI, and (**C**) zeta potential of empty GPs measured in the same media (n = 3). (**D**) Representative transmission electron microscopy (TEM) image of empty GPs suspended in water. (**E**) Particle size and (**F**) PDI of GPs measured in ultrapure water, 0.9% NaCl, DMEM, and RPMI medium after incubation at 37 °C for 24 h, 48 h, and 168 h. Data are presented as mean ± SD (n = 3). (**G**) Evaluation of protein encapsulation capacity: 100 µL of ovalbumin, myoglobin, or lysozyme was encapsulated within GPs at a concentration of 5 mg/mL. The particles were centrifuged, and the initial supernatants were analyzed using SDS-PAGE to visualize unencapsulated protein content. Protein standards (S) were included at a concentration of 2 mg/mL for reference.

Glucan particles (GPs) are the product of processed *Saccharomyces cerevisiae* yeast subjected to a series of alkaline and acidic washes. After purification, a comprehensive physicochemical analysis was conducted on GPs suspended in ultra-pure water, 0.9% NaCl solution, and DMEM and RPMI cell culture medium. In terms of size and size distribution, GPs exhibited consistent dimensions of approximately 4 µm across the various solvents, displaying minimal agglomeration (Table 2 and Figure 1A). The polydispersity index (PDI) values ranged between 0.2 and 0.4, indicating moderate polydispersity, which is typical and acceptable for microparticles derived from biological sources, such as the yeast *Saccharomyces cerevisiae* (Table 2 and Figure 1B). Since each microparticle essentially represents the outer shell of an individual yeast cell, the particle size—and consequently the size distribution—reflects the natural variability in the dimensions of the original yeast cells. The surface charge of GPs demonstrated a trend towards neutrality when suspended in 0.9% NaCl solution, DMEM or RPMI cell culture medium, as opposed to their suspension in water, where the surface charge was approximately −7 mV (Table 2 and Figure 1C). Furthermore, confirmation of the GPs’ size, shape, and structural composition was achieved through TEM analysis. This approach allowed us to visually ascertain the dimensions of GPs and observe their distinctive architecture, which comprises an outer shell and inner core (Figure 1D). These findings line up with the description provided by Soto et al. [12] and are consistent with subsequent research by Volpato and colleagues [25]. The stability of GPs in different media was assessed by monitoring changes in particle size (Figure 1E) and PDI (Figure 1F) over 24 h, 48 h, and 168 h of incubation at 37 °C. In water and 0.9% NaCl, a progressive decrease in particle size was recorded across all batches. This shrinkage is likely due to gradual erosion or disintegration of the glucan matrix, where the absence of stabilizing molecules may promote structural breakdown. In contrast, particle size increased over time in both DMEM and RPMI, with values rising consistently from 24 h through 168 h. This trend is attributed to swelling of the particles in nutrient-rich environments, as well as potential adsorption of proteins and salts present in the media. Additionally, the ionic strength and complex composition of these media may reduce electrostatic repulsion, contributing to soft aggregation or clustering, which manifests as an apparent size increase in measurements. Across all four solvents, a slight increase in PDI was observed over time. This rise reflects growing heterogeneity in the particle population, likely caused by asynchronous swelling, erosion, or aggregation processes occurring at different rates within each batch. Together, these results highlight the influence of medium composition on GP stability, where simple ionic or aqueous environments promote disassembly, while nutrient-rich or protein-containing media foster structural expansion and polydispersity.

In addition to their intrinsic immunostimulatory properties, GPs hold considerable value in vaccinology due to their ability to encapsulate a wide range of proteins. In this study, we evaluated the protein encapsulation efficiency of GPs using model proteins with varying molecular weights and isoelectric points. Encapsulation efficacy was assessed indirectly through SDS-PAGE analysis of the supernatants collected during the loading procedure (Figure 1G). Notably, regardless of the specific protein used, GPs consistently demonstrated effective internalization, corroborating previous findings reported in the literature [15]. SDS-PAGE was employed to validate these findings by estimating the residual unencapsulated protein. This electrophoretic method confirmed a high encapsulation efficiency, with loading efficiency (LE) consistently above 95% and loading capacity (LC) of approximately 5% (Figure S1).

Regarding the encapsulation of active molecules—specifically HBV antigens and CL097—the procedure was informed by the extensive experience of our research group in this area. In previous studies, we quantified the LE and LC of HBV antigens in GPs using ELISA, obtaining highly reproducible values of 98.8 ± 0.1% and 0.546%, respectively [26], and 98.5 ± 0.1% and 0.546% in a subsequent formulation [10]. Similar high encapsulation performance was also observed with structurally distinct antigens, such as the SARS-CoV spike protein, which achieved an LE of 98.5% [27]. For the immunostimulatory compound CL097, our previous work demonstrated an LE of 97% within GPs [27], further confirming the robustness of the platform across different types of cargo.

The encapsulation process involves hydrating lyophilized GPs with a minimal volume of solution containing the compound of interest. This facilitates passive diffusion of water and solute through the porous glucan shell, allowing for uniform internal distribution. The particles are subsequently re-freeze-dried to solidify the cargo retention. A tRNA solution is then applied to seal the porous openings, enhancing encapsulation by promoting molecular interactions between the loaded agents and the GP matrix. This sealing step serves not only to trap the compounds within the particle core but may also act as a stabilizing scaffold that improves overall loading efficiency and cargo retention [27].

### 3.2. Immunotoxicity Profile of GPs in RAW 264.7 Macrophages

**Figure 2:**
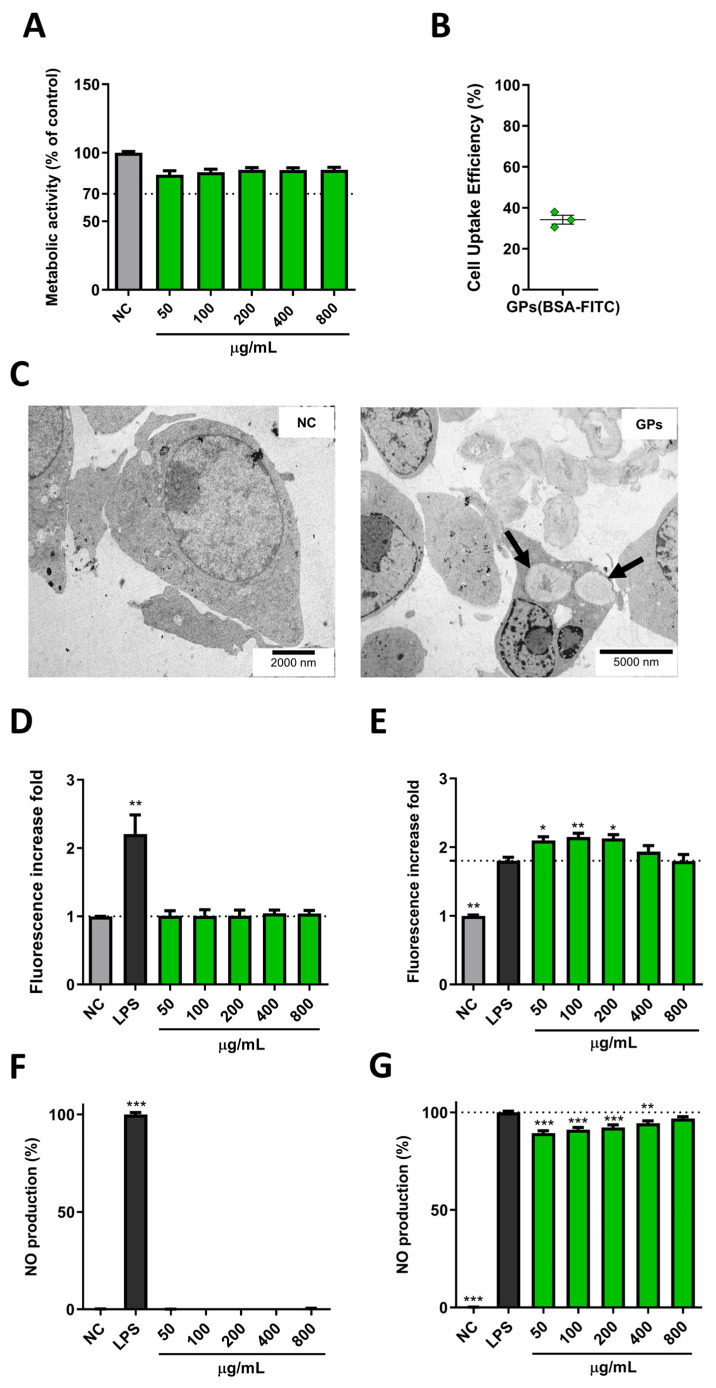
Assessment of GPs’ interactions with RAW 264.7 macrophages. (**A**) Cell viability, determined by the MTT assay after a 24 h GP incubation. Relative cell viability (%) was calculated compared to the negative control (n = 4). (**B**) Flow cytometry analysis of GPs (BSA-FITC) (5 µg/mL) internalization in RAW 264.7 macrophages at the 4 h time point. The percentage of fluorescent cells quantified to determine GP uptake (n = 3). (**C**) Electron microscopy images of RAW 264.7 macrophages. Untreated control cells (NC, left) and cells treated with GPs for 4 h (GPs, right). A representative image illustrates cellular ultrastructure with two internalized GPs within the cell’s cytoplasm (black arrows). (**D**) ROS production in RAW 264.7 cells exposed to GP formulations for 24 h. LPS (1 µg/mL) was used as a positive control, and unstimulated cells served as the negative control. The fold increase in fluorescence was calculated by comparing results with the negative control using DCFH-DA (n = 4, with three independent GP batches). (**E**) LPS-spiked ROS production in RAW 264.7 cells exposed to GP formulations and LPS (1 µg/mL) for 24 h. Fold increase in fluorescence was calculated by comparing results with LPS-stimulated cells using DCFH-DA (n = 3, with three independent GP batches). (**F**) NO production in RAW 264.7 cells exposed to GP formulations for 24 h. LPS (1 µg/mL) was used as a positive control, and unstimulated cells served as the negative control. NO production extrapolated from the calibration curve and compared to LPS-stimulated cells (100%) (n = 3, with three independent GP batches). (**G**) LPS-spiked NO production in RAW 264.7 cells exposed to GP formulations and LPS (1 µg/mL) for 24 h. NO production extrapolated from the calibration curve and compared to LPS-stimulated cells (100%) (n = 3, with three independent GP batches). Data are presented as mean ± SEM. Statistical analysis conducted using the Kruskal–Wallis test. *, **, and *** indicate significant differences compared to the control group with *p* ≤ 0.05, *p* ≤ 0.01, and *p* ≤ 0.001, respectively.

Macrophages play a pivotal role in orchestrating immune responses and contributing to various physiological processes crucial for maintaining homeostasis. To assess the cell viability of GPs on macrophages, we conducted an MTT reduction assay on the RAW 264.7 cell line (Figure 2A). The results demonstrate that GPs do not elicit cytotoxic effects on RAW 264.7 macrophages. Across all tested concentrations, cell viability remained consistently above 70%. This non-cytotoxic profile aligns with prior findings by Ren et al. [28], further substantiating the safety of yeast cell wall particles.

Flow cytometry analysis provided evidence of efficient GPs uptake by RAW 264.7 cells, with approximately a third (34.2 ± 3.7%) of the cells internalizing GPs within just a 4 h incubation period (Figure 2B). This rapid and effective particle uptake occurred even at a relatively low GP concentration, underscoring the cells’ proficiency in capturing these particles. The efficiency of particle uptake was further validated through TEM (Figure 2C). These microscopic images not only confirmed the internalization of GPs by RAW 264.7 cells but also revealed that a single cell could internalize more than one GP, emphasizing the remarkable efficiency of this process.

Reactive oxygen species (ROS), generated as byproducts of cellular metabolism, play a crucial role in cellular physiology. Evaluating ROS production in macrophages is critical in particle studies due to its implications for inflammatory responses and immune system activation. Remarkably, in our investigations, GPs did not induce elevated ROS production in RAW 264.7 cells following a 24 h incubation period (Figure 2D). Importantly, these conditions did not lead to cytotoxicity, signifying that GPs do not impose oxidative stress detrimental to cellular components. This suggests the biocompatible nature of GPs with macrophages, as controlled ROS levels indicate safer GP–cell interactions and an effective antioxidant defence. Excessive ROS generation could imply an overly active response, potentially resulting in oxidative stress, inflammation, genotoxicity and cell death [29]. Previous studies have linked ROS production to β-glucan nanoparticle (NP) size, where smaller nanoparticles triggered ROS production compared to larger counterparts [30].

Some particles possess antioxidant properties that modulate cellular ROS levels. To explore whether GPs could exert a protective role against oxidative stress, we conducted an ROS production assay by co-stimulating RAW 264.7 cells with LPS and GPs. Surprisingly, GPs not only failed to inhibit ROS but also exhibited a synergistic interaction with LPS, leading to increased ROS levels, especially at lower GP concentrations (Figure 2E). This phenomenon may be attributed to the concurrent activation of multiple immune pathways by LPS and GPs, an effect unachievable by GPs alone. GPs’ interactions with Dectin-1 or TLR 2/6 likely collaborate with LPS, known for its TLR 4 activation, triggering ROS production and pro-inflammatory responses [31].

The assessment of nitric oxide (NO) production in macrophages holds significant importance within particle studies, particularly when evaluating their potential immunomodulatory effects and biocompatibility. Notably, GPs did not demonstrate the capacity to induce NO production beyond basal levels (Figure 2F). The absence of NO production could be attributed to the particular glucan type used, as it has been suggested that the impact on macrophages and subsequent NO production hinges on the chemical composition of the glucans [32]. This is corroborated by the observed NO production induced by curdlan NPs [30,33].

Moreover, GPs exhibited a subtle yet significant inhibitory effect on NO levels when co-stimulated with LPS (Figure 2G). Importantly, this inhibition of NO by GPs did not affect cell viability. These findings are in line with previous studies investigating NO production and inhibition in RAW 264.7 macrophages exposed to β-glucans obtained from baker’s yeast [34,35]. The inhibition of NO production by GPs assumes significance, potentially serving as a mechanism through which to downregulate the inflammatory process. This regulatory role in limiting excessive inflammation and averting potential tissue damage underscores the delicate balance required for the effective resolution of the immune response. Recent studies have demonstrated the therapeutic potential of immunomodulatory biomaterials in reprogramming the immune microenvironment. Both Cai et al. and Qi et al. developed advanced hydrogels that promote M2 macrophage polarization and balance oxidative stress, supporting tissue repair [36,37]. While GPs differ structurally and compositionally from these systems, they share functional similarities in modulating inflammatory responses.

### 3.3. GPs’ Effects on Hemolysis and Immunomodulation in Human PBMCs

**Figure 3:**
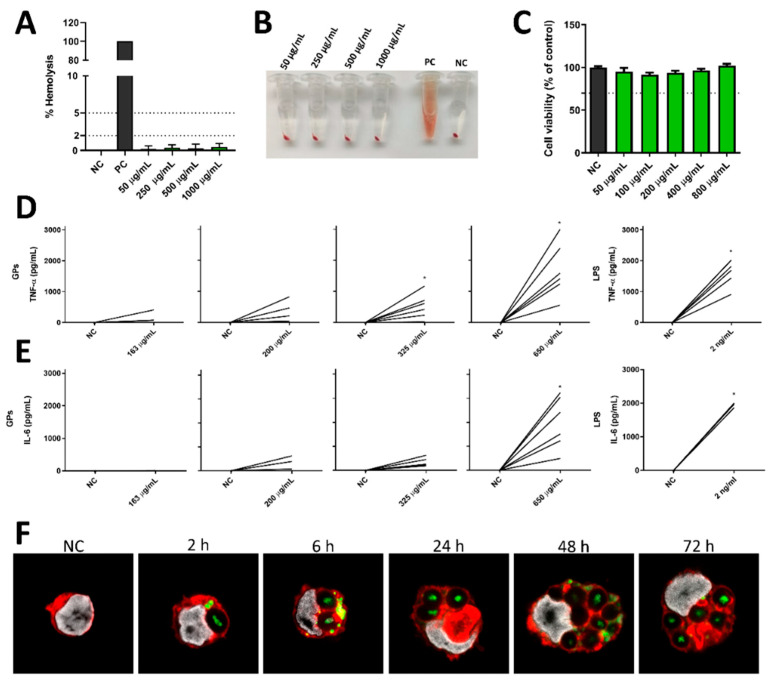
Evaluation of GPs’ effects on hemolysis and PBMCs. (**A**) The hemolytic effect of GPs on human whole blood compared to cells treated with Triton X-100 (PC—positive control) and cells treated with PBS (NC—negative control), with no hemolysis observed (n = 3). (**B**) Visual representation of tubes after the hemolysis assay, demonstrating the absence of hemolysis caused by GPs. (**C**) Impact of different concentrations of GPs on human PBMCs’ proliferation after 72 h of incubation (n = 3) assessed by MTT assay. (**D**) TNF-α and (**E**) IL-6 production by human PBMCs following 24 h of incubation with varying GPs concentrations (n = 6). Statistical analysis conducted using the Wilcoxon matched-pairs test. Significance denoted by * indicate *p*-values of ≤0.05. (**F**) Confocal microscopy images illustrating human monocytes treated with GPs (BSA-FITC) for different time periods. GPs’ localization shown in green fluorescence, cell nuclei stained in white using Hoechst 33342, and cellular membranes stained in red with Alexa Fluor^®^ 594. All data presented as mean ± SEM.

Hemolysis assessment is a critical aspect of material biocompatibility, serving as a key safety indicator for their potential use in biological systems. In this study, GPs demonstrated an excellent safety profile, as they exhibited no hemolytic effects across all tested concentrations (Figure 3A,B). The results consistently remained below the 5% hemolysis threshold, a crucial criterion for biomaterials to be deemed safe according to the American Society for Testing and Materials guidelines [38]. Interestingly, these findings align with those of Soares et al., who similarly demonstrated the absence of a hemolytic effect caused by β-glucan [39].

The immunomodulatory effects of GPs on human immune cells were evaluated using peripheral blood mononuclear cells (PBMCs) obtained from healthy donors. A proliferation assay was carried out to assess how these particles influence the immune system’s response, particularly in terms of enhancing or suppressing immune cell proliferation and metabolism. PBMCs were stimulated with GPs, and their metabolic activity was quantified after a 72 h incubation period using the MTT assay. Remarkably, it was observed that all tested concentrations of GPs exhibited non-cytotoxic effects on human PBMCs. However, these particles did not induce a proliferative response in PBMCs under the conditions tested (Figure 3C). This suggests that while GPs are well tolerated by immune cells, they do not significantly alter PBMC proliferation or metabolic activity.

TNF-α and IL-6 serve as key mediators of inflammation and immune responses. They not only stimulate the production of other immune-related molecules but also wield influence over the activity of various immune cells [3]. The quantification of TNF-α and IL-6 production by PBMCs serves as a valuable indicator of immune cell activation in response to GPs. Despite donor variability, our ELISA results consistently demonstrate that GPs elicit a concentration-dependent release of both TNF-α and IL-6 by human PBMCs, underscoring their immunomodulatory potential (Figure 3D,E). Elevated levels of these cytokines signify the activation of PBMCs in response to particle exposure. This activation has the potential to trigger the recruitment of additional immune cells to the site of exposure and to intricately influence immune cell behaviour, including the differentiation of T cells into distinct subsets, such as Th1 or Th2 cells, and the activation of macrophages. Remarkably, similar concentrations of IL-6 and TNF-α in human PBMCs were previously reported following incubation with 200 µg/mL of GPs [10]. Although individual donor responses varied in magnitude—as reflected in the SEM values—GPs consistently induced cytokine production above baseline across all donors. This underscores the platform’s reliability in eliciting immune activation in diverse human samples. Thus, the observed variability reflects biological diversity rather than inconsistency, supporting the translational robustness of GPs as immunomodulatory agents in a heterogeneous immune context.

The immunomodulatory potential of particles is closely linked to their internalization by immune cells. Thus, we sought to examine the capability of GPs to be internalized by human monocytes using confocal laser scanning microscopy (CLSM). Remarkably, within just 2 h of incubation of GPs loaded with BSA-FITC with monocytes, clear evidence of GPs’ internalization and their subsequent localization within the cytoplasm was observed (Figure 3F). Interestingly, over short time intervals, protein release from GPs into the cytoplasm was readily apparent. Contrary to the expected particle degradation within the cell over time, our observations revealed that GPs tended to accumulate within monocytes, to the extent that multiple particles were observed within each cell. This phenomenon highlights the robustness of GPs, as they resisted degradation for at least 72 h within human monocytes, suggesting their potential for sustained immunomodulatory activity and antigen release. The robust internalization of GPs by monocytes was expected, given that particulate β-glucans activate Dectin-1 receptor signalling. This receptor is broadly expressed on various myeloid cell populations and serves as a trigger for phagocytosis. Consequently, GPs are well documented as a selective delivery system with a strong affinity for phagocytic cells [40].

### 3.4. GPs’ Influence on Human moDCs’ Cytokine Release

**Figure 4:**
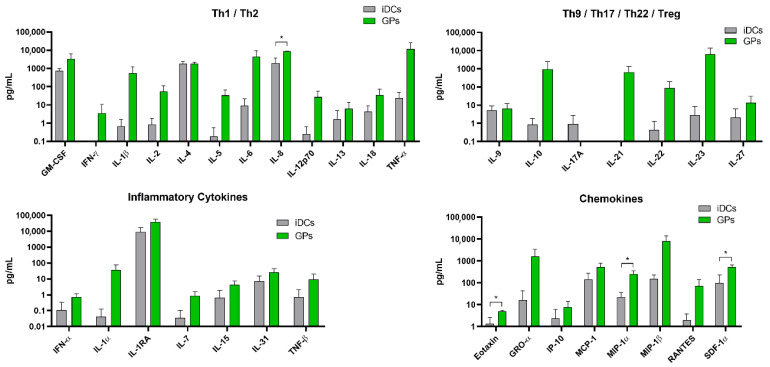
Evaluation of glucan particles (GPs)’ effects on human moDCs. Cytokine and chemokine concentrations were quantified in supernatants from human monocyte-derived dendritic cells (moDCs) treated with GPs (2.5 µg/mL) for 24 h. Statistical significance was assessed using multiple unpaired *t*-tests, with Bonferroni–Sidak correction applied to adjust for multiple comparisons (α = 0.05). Each analyte was analyzed independently, assuming no consistent standard deviation between groups (n = 4). Significance denoted by * indicate *p*-values of ≤0.05.

The impact of GPs on moDCs’ cytokine and chemokine production was thoroughly examined in this study. Notably, among the 34 cytokines and chemokines assessed, moDCs stimulated by GPs exhibited a statistically significant increase in the secretion of specific chemokines. These included IL-8, Eotaxin, MIP-1α, and SDF-1α (Figure 4 and Tables S1–S4).

When produced by activated DCs, these molecules function as chemoattractants, establishing a concentration gradient that effectively draws immune cells towards the vaccination site and amplifies the immune response [41]. This results in the development of a more robust protective state against the vaccine antigen. DC-secreted IL-8 seems to play a pivotal role in neutrophil recruitment and the maintenance of an inflammatory state [42], whereas SDF-1α is critical in kickstarting the immune response by boosting DC migration, maturation, and survival [43]. On the other hand, MIP-1α assists in recruiting naïve T cells to licensed DCs and aids in DC migration and T cell co-stimulation [44]. While certain other molecules, such as TNF-α, IL-10, or IL-21, showed an apparent increase upon GP stimulation, these elevations did not attain statistical significance due to the inherent variability among donors. Additionally, the relatively low GP concentration and the absence of antigenic stimulation may contribute to the observed lack of significant cytokine release.

### 3.5. GPs (HHC) Vaccination’s Influence on Humoral Immune Response

Conducting in vivo studies for efficacy verification is a crucial step following a series of in vitro tests. While in vitro experiments offer valuable insights into cellular and molecular interactions, they inherently have limitations that can only be addressed through in vivo research. In the context of this vaccination study, our objective was to evaluate the systemic reactions triggered by GPs encapsulating HBsAg, HBcAg, and CL097 (GPs (HHC)), specifically focusing on antibody generation, cytokine release, and immune cell activation.

**Figure 5:**
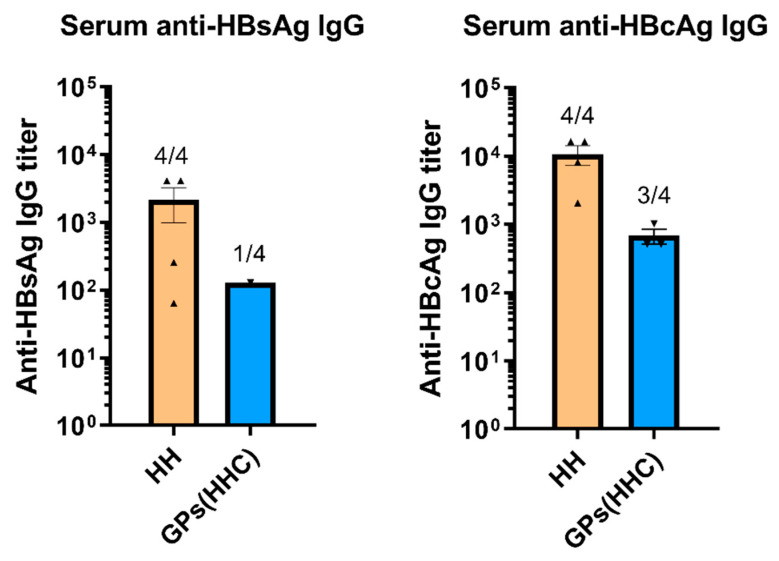
Serum levels of HBsAg-specific and HBcAg-specific IgG in vaccinated mice. Titers were defined as the highest plasma dilution resulting in an absorbance value twice as high as non-immune plasma. Data are presented as mean ± SEM.

The humoral immune response to vaccination was assessed by measuring serum levels of IgG specific to HBsAg and HBcAg in the vaccinated mice (Figure 5). In the HH group, all vaccinated mice produced detectable levels of IgG specific to both HBsAg and HBcAg. These IgG antibodies indicate a robust and consistent humoral immune response to the antigens. In contrast, the GPs (HHC) group exhibited variations in the humoral response. While some mice in this group did produce IgG specific to HBsAg and HBcAg, not all individuals mounted a meaningful IgG response. Furthermore, among those mice that did produce IgG, the antibody titers were frequently lower compared to the HH group. These results suggest that while vaccination with GPs (HHC) induced humoral immune responses in some mice, the response was less uniform and generally characterized by lower antibody titers compared to the HH group. This reduced humoral response to HBV antigen vaccination can be attributed to the abbreviated vaccination schedule consisting of only two immunizations. In fact, previous studies examining GPs encapsulating 1.5 µg of HBsAg administered three times subcutaneously achieved substantially higher IgG titers, with all animals developing protective titers [10]. While this study focused on total IgG production as a first indication of humoral response, we acknowledge that further investigations are necessary to evaluate the functional quality of the antibodies generated, such as their neutralizing capacity or affinity maturation. These aspects are particularly relevant for clinical translation and will be prioritized in future studies to better understand the protective potential of GP-based vaccine formulations.

### 3.6. Spleen Cell TNF-α Production upon Antigen Restimulation

**Figure 6:**
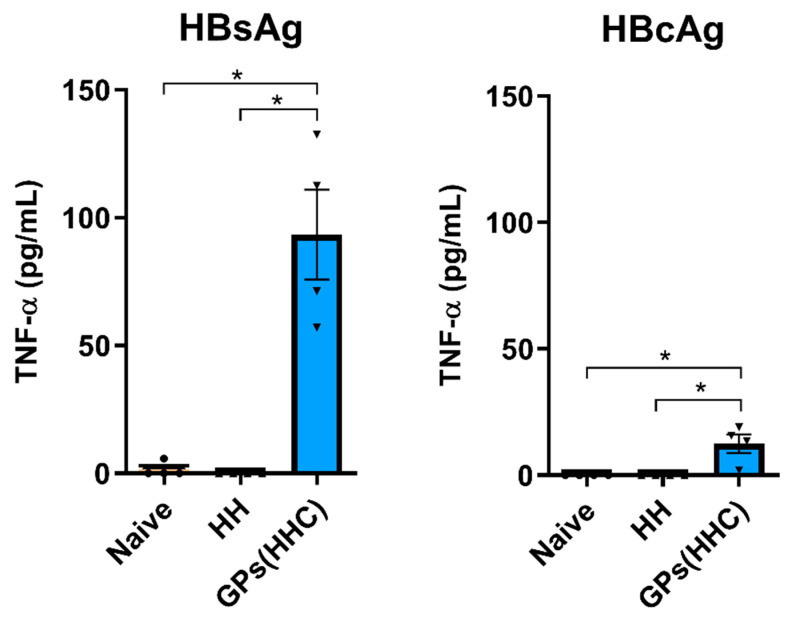
TNF-α levels in the supernatants of restimulated spleen cells measured through ELISA. Spleen cells were restimulated with HBsAg or HBcAg (1 µg/well), and TNF-α production was assessed after 96 h. Data are presented as mean ± SEM (n = 4). Statistical analysis was performed using the Kruskal–Wallis test. * indicates significant differences with *p* ≤ 0.05.

The production of TNF-α by spleen cells in response to antigen restimulation was evaluated to assess the cellular immune response in the vaccinated mice (Figure 6). In the HH group, none of the vaccinated mice produced detectable levels of TNF-α upon restimulation with either HBsAg or HBcAg. This observation suggests that TNF-α production was not significantly triggered by these antigens in the HH-vaccinated mice. Conversely, in the GPs (HHC) group, the response was distinct. Spleen cells from these mice produced significantly higher levels of TNF-α upon restimulation, compared to the naïve group, and notably, restimulation with HBsAg led to a more robust TNF-α response compared to restimulation with HBcAg. These findings indicate that the GPs (HHC) vaccination approach effectively stimulated TNF-α production in response to hepatitis B antigens, with HBsAg-specific cells being particularly prone to release TNF-α upon antigen restimulation. These results emphasize the differential immune responses induced by the two vaccination groups, with GPs (HHC) driving significant TNF-α production upon antigen restimulation, especially in response to HBsAg. Interestingly, Soares et al. achieved similar TNF-α concentrations upon HBsAg restimulation following a three-immunization schedule with GPs encapsulating HBsAg [10]. Additionally, since TNF-α drives the differentiation of CD4+ T cells toward the Th1 lineage [45], these results suggest that GPs (HHC) vaccination led to the maturation and activation of a significant Th1 population, which is critical for triggering the elimination of intracellular pathogens.

### 3.7. Effector Memory T Cell Phenotype After Spleen Cell Restimulation

**Figure 7:**
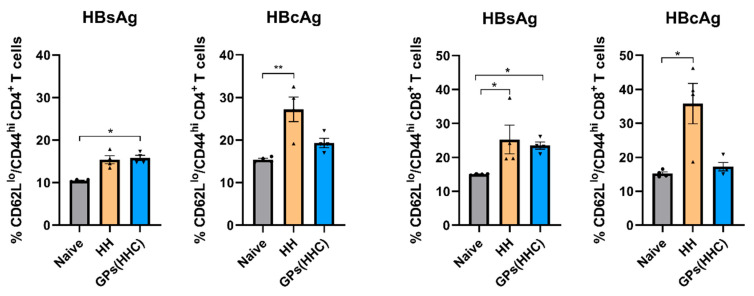
Effector memory phenotypic analysis of CD4^+^ and CD8^+^ T cells by flow cytometry after HBsAg or HBcAg (2 µg/well) restimulation for 6 h. The percentage of effector memory T cells (T_em_) was evaluated as CD62L^low^/CD44^high^ expressing T cells. Data are presented as mean ± SEM (n = 4). Statistical analysis was performed using the Kruskal–Wallis test. * and ** indicate significant differences compared to the control group with *p* ≤ 0.05 and *p* ≤ 0.01, respectively.

Effector memory T cells (T_em_) are vital components of the immune system, characterized by specific chemokine receptors and adhesion molecules necessary for rapid homing to inflamed tissues and prompt effector function [46]. These cells exhibit elevated expression of CD44, signifying that they received TCR signals, while concurrently showing diminished levels of CD62L, which restricts their migration to secondary lymphoid tissues, enabling them to extensively survey sites of infection within peripheral tissues [46,47]. In this flow cytometry study, we investigated the dynamic changes in the percentage of T_em_ cells within the CD4^+^ and CD8^+^ T cell populations following restimulation with HBsAg or HBcAg for 6 h (Figure 7).

In the HH-vaccinated group, restimulation with HBsAg did not induce a notable increase in the percentage of CD4^+^ T_em_ cells. Conversely, HBcAg restimulation in the HH group resulted in a significant elevation in the percentage of CD4^+^ T_em_ cells. These observations underscore the distinct impact of these antigens on CD4^+^ T cell responses within the HH-vaccinated mouse model. In contrast, the GPs (HHC)-vaccinated group exhibited a unique response pattern. Restimulation with HBsAg in the GPs (HHC) group led to a markedly higher percentage of CD4^+^ T_em_ cells compared to the naïve group. This highlights the specific and robust CD4^+^ T_em_ cell response induced by the GPs (HHC) vaccination strategy. However, HBcAg restimulation in the GPs (HHC) group did not significantly alter the percentage of CD4^+^ T_em_ cells, indicating a differential response to this antigen. Turning our attention to CD8^+^ T cells, the HH-vaccinated group displayed a substantial increase in the percentage of T_em_ cells after restimulation with either HBsAg or HBcAg. Conversely, the CD8^+^ T cells from the GPs (HHC) group primarily exhibited a significant elevation in the CD8^+^ T_em_ cell phenotype percentage when restimulated with HBsAg. Remarkably, results obtained from the GPs (HHC) group demonstrated a higher degree of reproducibility compared to the HH group. This reduced variation within the GPs (HHC) group may originate from the more controlled and consistent delivery of antigen and adjuvant to APCs when these molecules are encapsulated within GPs. In summary, these findings revealed the diverse effects of HBsAg and HBcAg on CD4^+^ and CD8^+^ T_em_ cell subsets as well as the distinct responses between the HH and GPs (HHC)-vaccinated groups.

### 3.8. Specific T Cell Cytokine Production Elicited by GPs (HHC) in C57BL/6 Mice

**Figure 8:**
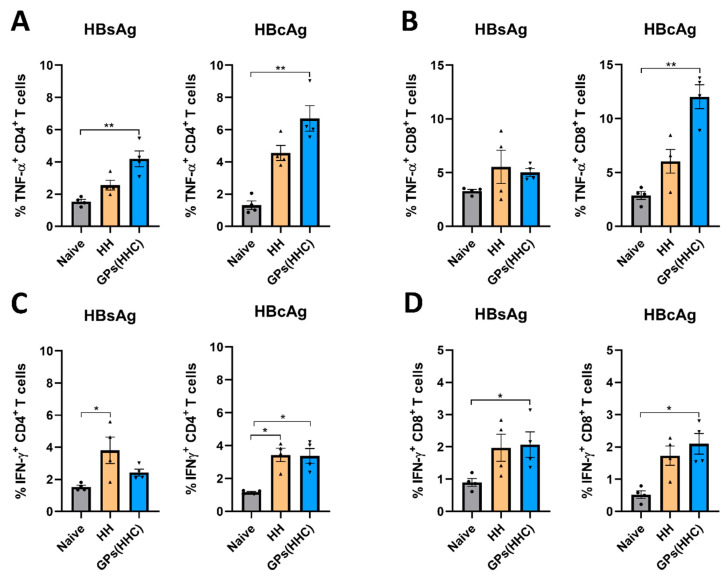
Evaluation of T cell immune responses elicited in C57BL/6 mice following vaccination with HH and GPs (HHC). Flow cytometry analysis was performed on CD4^+^ and CD8^+^ T cells after restimulation with HBsAg or HBcAg (2 µg/well). The percentage of positive TNF-α (**A**,**B**) and IFN-γ (**C**,**D**) expressing T cells was measured. Data are presented as mean ± SEM (n = 4). Statistical analysis was performed using the Kruskal–Wallis test. * and ** indicate significant differences compared to the control group with *p* ≤ 0.05, and *p* ≤ 0.01, respectively, compared to the control group.

TNF-α and IFN-γ are cytokines associated with T cell effector functions. TNF-α can induce apoptosis in infected cells, contributing to the elimination of HBV, while IFN-γ production is often associated with Th1 CD4^+^ T cells and cytotoxic CD8^+^ T cells. Flow cytometry analysis was performed on spleen CD4^+^ and CD8^+^ T cells after restimulation with HBsAg or HBcAg to quantify the antigen-specific immune response at the cellular level. The percentage of T cells expressing TNF-α and IFN-γ was measured (Figure 8A–D). In the GPs (HHC)-vaccinated group, TNF-α^+^ CD4^+^ T cells were present at a significantly higher frequency compared to the naïve group, regardless of whether they were stimulated with HBsAg or HBcAg. This suggests that the GPs (HHC) vaccination strategy promotes a robust CD4^+^ T cell response marked by elevated TNF-α expression. Remarkably, the GPs (HHC)-vaccinated group demonstrated a significant elevation in TNF-α^+^ CD8^+^ T cells exclusively upon HBcAg restimulation. These findings highlight the differential response of CD4^+^ and CD8^+^ T cell subsets to the antigens and the vaccination strategies employed. In the HH-vaccinated group, restimulation with HBsAg or HBcAg did not result in a significant increase in TNF-α^+^ T cells.

In the HH-vaccinated group, restimulation with HBsAg or HBcAg resulted in a significant increase in IFN-γ^+^ CD4^+^ T cells. Conversely, the GPs (HHC)-vaccinated group exhibited a higher frequency of IFN-γ^+^ CD4^+^ T cells compared to the naïve group, but this elevation was only observed upon HBcAg restimulation. This suggests that the GPs (HHC) vaccination approach primarily enhances IFN-γ expression in CD4^+^ T cells when exposed to HBcAg. Strikingly, the CD8^+^ T cell subset in the GPs (HHC)-vaccinated group demonstrated a substantial increase in IFN-γ^+^ cells in response to both HBsAg and HBcAg restimulation, reaching significant levels not observed in the HH group. This underscores the unique capacity of the GPs (HHC) vaccination strategy to provoke a potent IFN-γ response in CD8^+^ T cells.

The antigen-specific production of IFN-γ and TNF-α by Th1 cells is associated with cell-mediated immunity, which is particularly important against intracellular pathogens like HBV, since the activation of CD8^+^ T cells and macrophages eliminates both the pathogen and infected cells [48]. Additionally, IFN-γ and TNF-α have non-cytolytic effector functions by inhibiting HBV replication and contributing to cccDNA destabilization [49]. Through a comparative analysis of responses to HBsAg and HBcAg restimulation, valuable insights into the antigenic specificity of the immune response induced by the GPs (HHC) vaccine emerge. This comparison aids in discerning which antigens exhibit heightened immunogenicity and can trigger a more potent T cell response. Furthermore, it affirms the existence of antigen-specific memory within the T cell repertoire, shedding light on the vaccine’s efficacy and the immune system’s capacity to mount precise defences against distinct pathogens. These findings not only underscore the distinct cytokine responses orchestrated by different T cell subsets upon HBsAg and HBcAg restimulation but also highlight the effectiveness of the GPs (HHC) vaccination strategy in fostering a specific and robust TNF-α and IFN-γ response. This efficacy is particularly pronounced in the context of HBcAg, revealing the vaccine’s potential to elicit a vigorous and antigen-targeted immune defence. By focusing on HBcAg as their target, cytotoxic T lymphocytes (CTLs) assume a critical role in the elimination of HBV-infected cells. This function gains particular significance due to the high abundance of the core antigen within hepatocytes infected by HBV [50]. CTLs with specificity for HBcAg are essential in controlling the viral load, as they possess the capacity to distinguish and eliminate infected cells, thereby limiting the virus’s ability to replicate and propagate within the host. An additional advantage of targeting HBcAg lies in its relatively conserved nature compared to the surface antigen of HBV. While the latter can mutate, rendering it less susceptible to immune responses, the core antigen remains relatively stable [51]. Consequently, CTLs directed against HBcAg are less likely to encounter immune escape variants, making them effective against a broader spectrum of HBV strains. This strategic targeting of HBcAg by CTLs has far-reaching implications for the development of immune-based therapies against HBV infections.

Several studies have highlighted the growing interest in β-glucan-based delivery systems and their intrinsic immunomodulatory properties, particularly in the context of vaccine development and immune-targeted therapies. Several approaches have utilized modified yeast glucans or surface-bound particles to enhance delivery and immune response, often requiring additional components such as synthetic polymers, emulsifiers, or external adjuvants to achieve efficacy [52,53]. In contrast, the GPs employed in this study are biologically derived from *Saccharomyces cerevisiae* and preserve the native hollow shell structure, allowing for efficient protein encapsulation without the need for complex modifications or co-formulated adjuvants. These GPs function dually as delivery vehicles and immunostimulatory agents, primarily through recognition by Dectin-1 and other innate immune receptors, thereby promoting cytokine production and dendritic cell activation [27,54].

Recent advances in immunomodulatory biomaterials further emphasize the capacity of engineered systems to reshape immune responses. For example, Meng et al. developed a self-cascade hydrogel capable of scavenging ROS and glucose to alleviate oxidative stress, thereby promoting M2 macrophage polarization and tissue regeneration via suppression of NF-κB and IL-17 signaling [55]. Similarly, Qi et al. reported a hyperthermia-responsive hydrogel that effectively neutralized ROS and improved tissue oxygenation, which together modulated chronic inflammation and enhanced immune resolution [37]. While these synthetic systems offer refined control over immunological microenvironments, they often require complex fabrication and are limited to local delivery applications. In contrast, the glucan particles explored in the present study provide a naturally derived, off-the-shelf alternative with systemic and mucosal delivery potential. The use of tRNA-assisted encapsulation within GPs offers a simple, scalable method for protein loading, avoiding the complexity of nanoparticle systems that rely on chemical conjugation or layering [53]. Compared to traditional adjuvants such as alum or squalene emulsions—which have known limitations in terms of supply, sustainability, and immunological range—the glucan particle platform provides a robust and biocompatible option with intrinsic immunomodulatory activity [53,56]. Collectively, these features position glucan particles as next-generation self-adjuvant platforms that address key challenges in the development of safe, effective, and accessible vaccine delivery systems.

## 4. Conclusions

As the global effort to eradicate vaccine-preventable diseases intensifies, the demand for enhanced vaccination strategies becomes an essential topic. However, it is important, especially with innovative particulate delivery systems, to conduct thorough pre-clinical assessments to unveil any unforeseen toxicity profiles that could pose risks to human health. Potential side effects may arise from the physicochemical characteristics of these delivery systems, leading to unpredictable interactions with the immune system. This study provides a complete characterization of GPs, analyzing their physicochemical attributes, biocompatibility, and immunomodulatory potential. The results have clearly confirmed the biocompatibility of GPs, revealing their suitability for various applications. GPs have demonstrated an impressive capacity for efficient protein encapsulation and have exhibited the potential to modulate immune responses effectively. These findings have clarified their potential in the fields of immunotherapy and vaccine development, particularly in fighting infectious agents such as the hepatitis B virus. To unlock GPs’ full potential and translate these findings into practical therapies, further research is necessary.

## Figures and Tables

**Table 1:** Vaccination groups and schedule.

S.C. Route	HBsAg (µg/Dose)	HBcAg (µg/Dose)	GPs (µg/Dose)	CL097 (µg/Dose)	Priming, Boost, Euthanasia (Days)
Naive (n = 4)	---	---	---	---	21
HH (n = 4)	1.5	1.5	300	---	0, 14, 21
GPs (HHC) (n = 4)	1.5	1.5	300	5	0, 14, 21

**Table 2:** Summary of the mean particle size (µm), polydispersity index (PDI), and zeta potential (mV) of empty GPs suspended in ultrapure water, 0.9% NaCl, DMEM, and RPMI. Data are presented as mean ± standard deviation (SD) (n = 3).

	H_2_O	0.9% NaCl	DMEM	RPMI
Size (µm)	4565 ± 692	4945 ± 274	4875 ± 419	3987 ± 772
PDI	0.203 ± 0.120	0.278 ± 0.163	0.288 ± 0.074	0.358 ± 0.135
Zeta Potential (mV)	−6.94 ± 0.81	−1.19 ± 0.19	−1.07 ± 0.43	−3.49 ± 0.42

## Data Availability

The raw data supporting the conclusions of this article will be made available by the authors on request.

## Supplementary Materials

The following supporting information can be downloaded at: https://www.mdpi.com/article/10.3390/pharmaceutics17081032/s1.
