# Supplementary material for: Yeast-Derived Glucan Particles: Biocompatibility, Efficacy, and Immunomodulatory Potential as Adjuvants and Delivery Systems
Source: Pharmaceutics. 2025 Aug 8;17(8):1032. doi: 10.3390/pharmaceutics17081032 (PMC12388970; doi:10.3390/pharmaceutics17081032)
Supplement: Supplementary file 1 [file pharmaceutics-17-01032-s001.zip › pharmaceutics-3793922-supplementary.pdf]

Supplementary Material

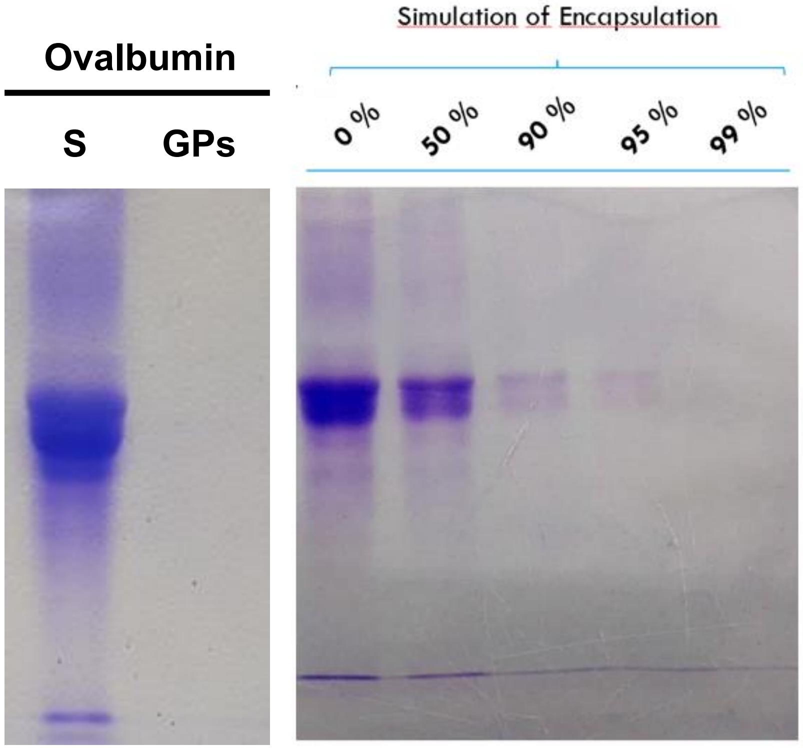

Figure S1: Evaluation of protein encapsulation capacity in GPs. Glucan particles were loaded with 100 μL of ovalbumin. Following encapsulation, particles were centrifuged, and the resulting supernatants and wash fractions were analyzed by SDS-PAGE to assess unencapsulated protein content. Protein standards (S) were included at 2 mg/mL for reference. To estimate the lower detection limit of the assay, control samples with known concentrations of ovalbumin were run in parallel. The absence of detectable protein bands in the supernatants and washes suggests that at least 95 % of the protein was successfully encapsulated.

Table S1: Individual Th1/Th2 cytokine concentrations (pg/mL) were measured using a 34-plex immunoassay. The table includes raw values for four independent donors under control and glucan particle (GP)-treated conditions (2.5 µg/mL for 24 h), along with calculated group means and standard deviations (SD).

pg/mL	iDCs				GPs				iDCs Mean	iDCs SD	GPs Mean	GPs SD
**GM-CSF**	755.1	1036.8	626.46	590.86	1142.78	700.31	5127.92	6759.02	752.305	202.3583	3432.508	2980.373
**IFN-γ**	0	0	0	0	0	0	0	14.28	0	0	3.57	7.14
**IL-1β**	1.88	0	0.88	0	7.25	17.33	607.1	1520.2	0.69	0.895247	537.97	712.3399
**IL-2**	1.87	0	1.56	0	0	9.32	109.17	100.26	0.8575	0.998211	54.6875	58.00612
**IL-4**	1632.92	1210.92	2483.17	1965.83	1458.84	1532.81	2244.46	2068.89	1823.21	537.5863	1826.25	389.3888
**IL-5**	0	0	0.76	0	6.53	3	64.81	60.88	0.19	0.38	33.805	33.60178
**IL-6**	2.64	3.05	2.09	28.29	258.83	58.77	6498.98	10,500	9.0175	12.85435	4329.145	5085.64
**IL-8**	1349.91	285.28	1388.85	4637.45	8800	8675	8575	8856	1915.373	1885.372	8726.5	126.1969
**IL-12p70**	0.25	0	0.79	0	0.58	2.51	59.97	44.3	0.26	0.372469	26.84	29.9109
**IL-13**	6.56	0	0	0	0	4.5	3.49	17.2	1.64	3.28	6.2975	7.519665
**IL-18**	3.21	0	3.07	11.04	8.75	3.21	37.48	88.28	4.33	4.712218	34.43	38.91562
**TNF-α**	13.3	8.92	59.99	13.65	146.18	101.38	23400	2340.0	23.965	2,411,289	11,761.89	13,438.54

Table S2: Individual Th9/Th17/Th22/Treg cytokine concentrations (pg/mL) were measured using a 34-plex immunoassay. The table includes raw values for four independent donors under control and glucan particle (GP)-treated conditions (2.5 µg/mL for 24 h), along with calculated group means and standard deviations (SD).

pg/mL	iDCs				GPs				iDCs Mean	iDCs SD	GPs Mean	GPs SD
**IL-9**	6.13	9.48	5.32	0	13.97	3.73	0	8.12	5.2325	3.925705	6.455	6.00944
**IL-10**	0.31	0.86	0	2.32	32.33	8.72	473.13	3222.56	0.8725	1.028441	934.185	1540.461
**IL-17A**	3.65	0	0	0	0	0	0	0	0.9125	1.825	0	0
**IL-21**	0	0	0	0	5.89	8.14	1131.88	1411.87	0	0	639.445	739.1596
**IL-22**	1.77	0	0	0	0	2.89	126.63	220.86	0.4425	0.885	87.595	106.6632
**IL-23**	11.26	0	0	0	10.97	59.33	13,969.49	10,910.29	2.815	5.63	6237.52	7269.986
**IL-27**	8.41	0	0	0	0	15.56	0	37.89	2.1025	4.205	13.3625	17.9215

Table S3: Individual inflammatory cytokine concentrations (pg/mL) were measured using a 34-plex immunoassay. The table includes raw values for four independent donors under control and glucan particle (GP)-treated conditions (2.5 μg/mL for 24 h), along with calculated group means and standard deviations (SD).

pg/mL	iDCs				GPs				iDCs Mean	iDCs SD	GPs Mean	GPs SD
**IFN-α**	0.44	0	0	0	0.9	1.17	0	0.7	0.11	0.22	0.6925	0.500225
**IL-1α**	0.17	0	0	0	1.29	3.15	61.69	81.23	0.0425	0.085	36.84	40.77096
**IL-1RA**	20,550.21	4345.65	7080.9	4512.07	27,156.12	44,971.79	63,422.64	20,098.66	9122.208	7720.86	38,912.3	19404.77
**IL-7**	0.14	0	0	0	0.81	0.26	0.62	1.83	0.035	0.07	0.88	0.673152
**IL-15**	2.57	0	0	0	8	3.64	0.84	4.75	0.6425	1.285	4.3075	2.960826
**IL-31**	18.92	5.44	0	4.49	23.26	35.77	0	42.65	7.2125	8.157603	25.42	18.75133
**TNF-β**	2.84	0	0	0	8.29	4.59	0.19	24.67	0.71	1.42	9.435	10.6827

Table S4: Individual chemokines concentrations (pg/mL) were measured using a 34-plex immunoassay. The table includes raw values for four independent donors under control and glucan particle (GP)-treated conditions (2.5 μg/mL for 24 h), along with calculated group means and standard deviations (SD).

pg/mL	iDCs				GPs				iDCs Mean	iDCs SD	GPs Mean	GPs SD
**Eotaxin**	0.92	0.43	0.75	3.21	5.68	4.48	4.17	5.26	1.3275	1.271335	4.8975	0.694568
**GRO-α**	0.86	3.25	4.57	56.54	130.06	47.61	3087.35	3268.72	16.305	26.86724	1633.435	1785.404
**IP-10**	0.33	1.12	0.03	7.82	8.08	2.02	4.35	16.5	2.325	3.692068	7.7375	6.352524
**MCP-1**	52.09	214.99	19.45	290.76	425.9	215.99	771.44	693.73	144.3225	129.7919	526.765	254.6226
**MIP-1α**	13.71	9.08	29.3	38.69	333.41	307.32	228.04	114.61	22.695	13.73031	245.845	98.29623
**MIP-1β**	125.68	72.33	185.11	234.05	2808.85	6573.18	7375	16,644.61	154.2925	70.35032	8350.41	5876.865
**RANTES**	0.54	0.66	2.09	4.52	24.19	22.98	83.68	160.26	1.9525	1.850826	72.7775	64.83977
**SDF-1α**	48.42	0	61.87	287.6	476.29	394.07	683.67	529.43	99.4725	128.2017	520.865	121.9873
